# Mechanisms underlying the EEG biomarker in Dup15q syndrome

**DOI:** 10.1186/s13229-019-0280-6

**Published:** 2019-07-03

**Authors:** Joel Frohlich, Lawrence T. Reiter, Vidya Saravanapandian, Charlotte DiStefano, Scott Huberty, Carly Hyde, Stormy Chamberlain, Carrie E. Bearden, Peyman Golshani, Andrei Irimia, Richard W. Olsen, Joerg F. Hipp, Shafali S. Jeste

**Affiliations:** 1 Roche Pharma Research and Early Development, Neuroscience, Ophthalmology and Rare Diseases, Roche Innovation Center Basel, Basel, Switzerland; 2 Center for Autism Research and Treatment, University of California Los Angeles, Semel Institute for Neuroscience, Los Angeles, CA 90024 USA; 3 Department of Psychology, University of California Los Angeles, 3423 Franz Hall, Los Angeles, CA 90095 USA; 4 Departments of Neurology, Pediatrics and Anatomy & Neurobiology, The University of Tennessee Health Science Center, 855 Monroe Ave., Link, Memphis, TN 415 USA; 5 McGill University, MUHC Research Institute, 5252, boul. de Maisonneuve Ouest, 3E.19, Montreal, QC H4A 3S5 Canada; 6 Genetics and Genome Sciences, UConn Health, 400 Farmington Avenue, Farmington, CT 06030-6403 USA; 7 Department of Psychiatry and Biobehavioral Sciences and Department of Psychology, University of California Los Angeles, Suite A7-460, 760 Westwood Plaza, Los Angeles, CA 90095 USA; 8 Department of Neurology and Psychiatry, David Geffen School of Medicine, 710 Westwood Plaza, Los Angeles, CA 90095 USA; 9 Leonard Davis School of Gerontology, University of Southern California, 3715 McClintock Ave., Suite 228C, California, Los Angeles 90089 USA; 10 Department of Molecular and Medical Pharmacology, David Geffen School of Medicine at UCLA, California, Los Angeles 90095 USA

**Keywords:** Dup15q syndrome, GABA, UBE3A, Biomarkers, Autism, EEG, Neurodevelopmental disorders, GABRA5, GABRB3, GABRG3

## Abstract

**Background:**

Duplications of 15q11.2-q13.1 (Dup15q syndrome), including the paternally imprinted gene *UBE3A* and three nonimprinted gamma-aminobutyric acid type-A (GABA_A_) receptor genes, are highly penetrant for neurodevelopmental disorders such as autism spectrum disorder (ASD). To guide targeted treatments of Dup15q syndrome and other forms of ASD, biomarkers are needed that reflect molecular mechanisms of pathology. We recently described a beta EEG phenotype of Dup15q syndrome, but it remains unknown which specific genes drive this phenotype.

**Methods:**

To test the hypothesis that *UBE3A* overexpression is not necessary for the beta EEG phenotype, we compared EEG from a reference cohort of children with Dup15q syndrome (*n* = 27) to (1) the pharmacological effects of the GABA_A_ modulator midazolam (*n* = 12) on EEG from healthy adults, (2) EEG from typically developing (TD) children (*n* = 14), and (3) EEG from two children with duplications of paternal 15q (i.e., the *UBE3A*-silenced allele).

**Results:**

Peak beta power was significantly increased in the reference cohort relative to TD controls. Midazolam administration recapitulated the beta EEG phenotype in healthy adults with a similar peak frequency in central channels (*f* = 23.0 Hz) as Dup15q syndrome (*f* = 23.1 Hz). Both paternal Dup15q syndrome cases displayed beta power comparable to the reference cohort.

**Conclusions:**

Our results suggest a critical role for GABAergic transmission in the Dup15q syndrome beta EEG phenotype, which cannot be explained by *UBE3A* dysfunction alone. If this mechanism is confirmed, the phenotype may be used as a marker of GABAergic pathology in clinical trials for Dup15q syndrome.

**Electronic supplementary material:**

The online version of this article (10.1186/s13229-019-0280-6) contains supplementary material, which is available to authorized users.

## Background

Duplications and triplications of 15q11.2-q13.1 (Dup15q syndrome) are highly penetrant for intellectual disability (ID), autism spectrum disorder (ASD), delayed development, and epilepsy [1–4]. Dup15q syndrome is often considered the most recurrent copy number variant implicated in ASD [5]. Several genes in this region impact early brain development, namely synaptic function and inhibitory neurotransmission [6–8]. The relative contributions of these genes to Dup15q syndrome pathology are poorly understood. However, allele-specific expression in neurons (i.e., maternal or paternal imprinting) may allow their contributions to be elucidated by examining maternal and paternal duplications separately.

Parent-of-origin modulates the Dup15q syndrome clinical phenotype. Children with maternal duplications present with a more severe clinical phenotype and greater likelihood of ASD and ID [9]. This discrepancy is likely due to paternal imprinting of *UBE3A* in most neurons [10, 11], a gene implicated in neurodevelopmental disorders [12, 13] that encodes a ubiquitin-protein ligase and regulates synaptic development [6, 7, 14]. Two 15q duplication types exist: interstitial and isodicentric duplications [2]. Interstitial duplications manifest as extra copies of 15q11.2-q13.1 found within chromosome 15 and generally result in partial trisomy or, less commonly, partial tetrasomy. Isodicentric duplications are extra copies of 15q11.2-q13.1 ligated end-to-end as a supernumerary chromosome, resulting in partial tetrasomy and conferring a more severe clinical phenotype [2]. In children with maternal interstitial and isodicentric duplications, upwards of 50% and 80% meet the criteria for ASD, respectively [2–4, 15–17].

To guide targeted treatments of Dup15q syndrome and other forms of ASD, biomarkers are needed that reflect a molecular or circuit level treatment response [18]. Such mechanism-based biomarkers may serve as surrogate endpoints in clinical trials whose short durations preclude observation of long-term behavioral changes. They also can serve as quantifiable measures of drug target engagement which, in turn, can inform decisions around continuation in a trial. Dup15q syndrome is characterized by a distinct electroencephalogram (EEG) phenotype that likely reflects molecular pathology [4, 15]. Recently, our group quantified this EEG phenotype as spontaneous beta band (12-30 Hz) oscillations in children with Dup15q syndrome, none of whom were taking benzodiazepines or other medications known to induce beta activity [19]. The Dup15q EEG phenotype is thus a promising biomarker that may quantify disease pathophysiology or index drug target engagement in the development of Dup15q syndrome treatments.

Proper application of the Dup15q syndrome biomarker will crucially depend on understanding which genes and which aspects of Dup15q syndrome pathophysiology the biomarker reflects. Several 15q genes have been linked to the disease etiology, including the paternally imprinted gene *UBE3A* and a cluster of non-imprinted gamma-aminobutyric acid type-A (GABA_A_) receptor β3, α5, and γ3 subunit genes [2]. As evidence of its involvement in neurodevelopmental disorders, *UBE3A* is the causative gene of Angelman syndrome [20], a disorder resulting in the majority of cases from deletion of maternal 15q11.2-q13.1 [21] and characterized by phenotypic overlap with Dup15q syndrome [22–27]. A role for *UBE3A* in Dup15q syndrome pathophysiology is thus likely. However, because *UBE3A* is only expressed from the maternal allele in human neurons [10, 11], *UBE3A* dysfunction is unlikely to be responsible for the clinical manifestations of cases of paternal 15q duplication [4]. Phenotypes common to both paternal and maternal duplications, on the other hand, would be best explained by biallelically expressed, nonimprinted genes. The most likely non-imprinted candidate genes within the duplication are a cluster of GABA receptor subunit genes including *GABRB3*, *GABRA5*, *and GABRG3*. These GABA_A_ receptor genes encode the β3, α5, and γ3 subunits, respectively, and they have been linked to epilepsy and ASD in both patients and animal models [28–32]. A potential role for the *GABRB3*/*GABRA5*/*GABRG3* gene cluster in the Dup15q syndrome EEG phenotype is emphasized by a similarity between the Dup15q syndrome beta EEG pattern [19] and the well-documented phenomenon of beta oscillations induced by GABA_A_-modulating compounds (e.g., benzodiazepines) in the human EEG [33]. Furthermore, in children with Angelman syndrome, patients with deletions of 15q11.2-q13.1 that encompass the *GABRB3*/*GABRA5*/*GABRG3* gene cluster (i.e., the genetic converse of Dup15q syndrome) feature reduced beta power [27] and a more severe clinical phenotype [34–37] relative to patients with etiologies not encompassing the GABA_A_ receptor genes. The foregoing evidence from Angelman syndrome underscores the influence of GABA_A_ receptor genes on both clinical phenotype and beta EEG phenotype in 15q disorders.

To infer the extent to which involvement of *UBE3A* or the *GABRB3*/*GABRA5*/*GABRG3* gene cluster is necessary or sufficient for the beta EEG phenotype in Dup15q syndrome, we performed three studies. First, to confirm that our earlier characterization of the beta EEG phenotype still holds in a larger Dup15q syndrome sample, we compared beta power in healthy, typically developing (TD) children to beta power in a reference cohort of children with Dup15q syndrome featuring both interstitial and isodicentric duplications. Next, to test the hypothesis that GABAergic dysfunction is sufficient to produce the beta EEG phenotype, we compared this phenotype in Dup15q syndrome to beta oscillations pharmacologically induced by the GABA_A_ modulator midazolam in healthy adults. Finally, to test the hypothesis that *UBE3A* dysregulation is necessary for the beta EEG phenotype, we compared two cases of paternal Dup15q syndrome to the aforementioned reference cohort of children with Dup15q syndrome. Each of the studies described above is motivated by the overarching goal of improving clinical trials in Dup15q syndrome. As we begin to understand the mechanism underlying this EEG biomarker, we can apply it rationally in pharmacological trials as an index of treatment response or drug target engagement.

## Methods

See Additional file 1: Methods and Materials for an extended description of the methods.

### Recruitment and EEG acquisition

To test our predictions outlined above, we analyzed spontaneous EEG recordings from (1) a reference cohort of *n* = 27 children with Dup15q syndrome, (2) a control cohort of *n* = 14 children with typical development, (3) two children with paternal duplications of 15q11-q13, and (4) *n* = 12 healthy adult volunteers challenged with 5 mg of midazolam. The reference cohort and TD control cohort included *n* = 13 and *n* = 9 participants, respectively, from a previous study of the Dup15q syndrome beta EEG phenotype by Frohlich and colleagues [19]. Clinical EEGs from both children with paternal Dup15q syndrome were examined in a previous study by Urraca and colleagues [4], and one child’s (801-005) research EEG was also examined in Frohlich and colleagues [19]. Recruitment and data acquisition are detailed for each below.

### Dup15q syndrome reference cohort

Because Dup15q syndrome is a rare disease with a 1 in 10,000 prevalence rate [38], we partnered with a patient advocacy group, the Dup15q Alliance, and collected data from children at two national family conferences to increase our sample size in accordance with the University of California, Los Angeles (UCLA) Institutional Review Board (IRB). We recruited children of all ages and developmental abilities in order to capture the most clinically representative sample. Parents of participants provided informed written consent prior to the start of study activities. We cautiously excluded data from participants with confounding factors such as epilepsy and antiepileptic medications that act on GABAergic transmission (to our knowledge, at least three participants included in our analysis later developed seizures after EEG data were acquired). Furthermore, we excluded participants with confirmed paternal duplications from the reference cohort. Because genetic reports obtained from parents generally did not contain parent-of-origin data, only two cases of maternal parent-of-origin were confirmed in the reference cohort. Nonetheless, it is overwhelmingly likely that the majority of our reference cohort consists of children with maternal duplications, given both the fact that maternal duplications are roughly twice as common as paternal duplications and roughly 2.5 times more penetrant for ASD and developmental delay as paternal duplications [39]. Reference cohort data presented here are from *n* = 27 participants with Dup15q syndrome (*n* = 13 interstitial, *n* = 14 isodicentric). See Table 1 for details of the reference cohort age and developmental quotient (DQ).

**Table 1:** Dup15q syndrome reference cohort. Cognitive ability is reported as developmental quotient derived (DQ) from age-appropriate developmental scales. Calculations of mean and standard deviation (SD) for DQ scores ignore missing data reported in rows ‘missing DQ’ row

	Interstitial	Isodicentric	Total
Dup15q reference cohort	13	14	27
Dup15q age (months, mean ± SD)	80.0 ± 42.7 (min = 9, max = 175)	56.9 ± 37.0 (min = 18, max = 156)	68.0 ± 40.8 (min = 9, max = 175)
Dup15q DQ (mean ± SD)	45.2 ± 23.7	35.8 ± 16.8	40.5 ± 20.6
Dup15q missing DQ	1	2	3
Dup15q ADOS calibrated severity score	6.13 ± 2.59	7.33 ± 1.44	6.85 ± 2.01
Dup15q missing ADOS	5	2	7
TD sample	N/A	N/A	14
TD age (months, mean ± SD)	N/A	N/A	55.0 ± 28.5 (min = 16, max = 111)
TD DQ (mean ± SD)	N/A	N/A	118 ± 15.5

High density (HD) EEG data were acquired at a sampling rate of 500 Hz using 129 channel vertex-referenced EGI geodesic nets with Ag/AgCl electrodes (Electrical Geodesics, Inc., Eugene, OR, USA). Full details of the data acquisition are found in a previous publication [19].

### TD control group

To confirm high beta power in the Dup15q syndrome reference cohort, we examined awake-state spontaneous EEG data from TD children (*n* = 14) recruited through UCLA. The control group did not significantly differ in age from the Dup15q syndrome reference cohort (*p* = 0.29, *t* = 1.06). All EEG data were recorded at UCLA. Recruitment, parental consent, and EEG protocol were identical to that described above for Dup15q syndrome. See Table 1 for age and DQ details.

### Paternal Dup15q syndrome case studies

Cases of paternal Dup15q syndrome are observed less frequently than maternal Dup15q syndrome, owing to a much milder clinical presentation [4, 39]. This fact impedes both detection and recruitment. We obtained EEG from two children with paternal duplications (see Table 2). Both paternal duplication participants have previously been clinically described in a study of individuals with interstitial Dup15q syndrome [4]. Thus, they are referred to here by their IDs from the previous publication. The first participant (801-005) was a boy aged 13 years (161 months) with paternally derived interstitial Dup15q syndrome recruited through UCLA at a Dup15q Alliance family conference. Awake-state spontaneous EEG data were recorded from 801-005 using the high-density EGI system and protocol described above. The second participant (801-015) was a girl aged 8 years (96 months) with paternally derived interstitial Dup15q syndrome. Awake-state spontaneous EEG data were collected from 801-015 at LeBonheur Children’s Hospital (LCH) in Memphis, Tennessee (sampling rate = 512 Hz). Data were referenced to average prior to importing. We excluded ear channels, yielding 19 channels (standard 10-20 system) for analysis.

**Table 2:** Phenotype, duplication, and EEG details of participants with paternal Dup15q syndrome. Participant 801-005 was a 13-year-old boy with paternal Dup15q syndrome. Participant 801-015 was an 8-year-old girl with paternal Dup15q syndrome. Both participants had interstitial duplications and were diagnosed with attention deficit hyperactivity disorder (ADHD). Neither participant had seizures or a diagnosis of ASD. However, 801-005 met criteria for ASD on the diagnostic observation schedule (ADOS) administered at the time of EEG (calibrated severity score = 7); this was likely due to working memory and attentional deficits related to ADHD [4]. Both participants had similar DQs, though 801-005, but not 801-015, had a DQ measured below the threshold for ID

ID	Dup type	Dup size	Age	Gender	Seizures	ASD	ADHD	Full DQ	EEG
801-005	Interstitial	5.7 Mb	161 months	M	No	No	Yes	67	Research
801-015	Interstitial	5.0 Mb	96 months	F	No	No	Yes	77	Clinical

### Midazolam pharmaco-EEG

To assess the similarity of the beta EEG phenotype in Dup15q syndrome to beta oscillations pharmacologically induced with a GABA_A_ positive allosteric modulator (PAM), we examined 19-channel EEG (sampling rate = 256 Hz) from *n* = 12 healthy adult controls challenged with the benzodiazepine compound midazolam. Midazolam is a nonselective GABA_A_ PAM (i.e., benzodiazepine) that binds to the GABA_A_ receptor, increasing the conductance of the receptor when the channel is opened by GABA [40]. The study protocol was approved by the National Research Ethics Service (NRES) committee. These data were acquired as part of a Roche sponsored trial (WP29393). The study also investigated other endpoints and conditions that are not reported here.

### EEG preprocessing

Raw data were imported to MATLAB (The MathWorks, Inc., Torrance, California) for data processing. Data were bandpass filtered 1–45 Hz (FIR filter) and artifact reduced using a combination of manual artifact selection and independent component analysis (ICA). We excluded 46 “skirt channels” from HD EEG data that are particularly sensitive to noise and muscle artifact (see Additional file 1: Figure S1), leaving 83 channels for processing and analysis. Manual artifact selection identified technical artifacts and gross physiological artifacts for exclusion. Noisy channels were also marked for interpolation at this stage, and datasets for which the number of noisy channels exceeded the square root of the total number of imported channels were excluded from the analysis. A minimum of 60 s of clean data was analyzed for each participant. ICA was performed with the FastICA algorithm [41, 42]. Components corresponding to stereotyped physiological artifacts (e.g., blinks, saccades, neck movement) were subtracted from EEG data. Following artifact reduction, bad channels were spline-interpolated. Data were averaged referenced prior to the wavelet transform. In contexts where HD EEG were compared directly to 19-channel EEG, we spatially interpolated HD EEG to 19 channels corresponding to the 10-20 montage coordinates prior to the wavelet transform. See Additional file 1: Table S1 for number of bad channels, artifact components, and length of good data for each cohort and paternal duplication case.

### Frequency transform and analysis

Data were frequency transformed using Morlet wavelets [43]. A total of 54 Morlet wavelet kernels were used with logarithmically spaced frequencies from 2 to 45 Hz (12 wavelets per octave) and with a spectral smoothing of 1/3 octave. Next, elements of the time-frequency representation corresponding to excluded data were removed. Datasets were discarded if their time-frequency representation contained fewer than 20 valid (i.e., non-excluded) time windows for the 2 Hz wavelet transform. We estimated spectral power by averaging power values of successive 3/4-overlapping temporal windows of continuous clean data in time-frequency representations. This gave a single estimation of spectral power at each of either 83 (HD EEG) or 19 (10-20 system) channels and 54 frequency bins.

We smoothed frequency output in half-octave bins and normalized power at each bin by log_2_(Hz) (i.e., octave) to yield power spectral densities (PSDs). We then computed PSDs using log_2_(Hz) and plotted PSDs in a logarithmic space to account for the logarithmic nature of electrophysiological signals [44]. Our analysis used absolute power because relative power measurements are vulnerable to normalization artifacts. For example, large theta oscillations present in several of our participants artifactually reduce beta band power when relative power is computed. In instances where channel-averaged power was reported, we first averaged across channels before log-scaling PSDs and then averaging across participants.

To compare PSDs from paternal Dup15q syndrome cases to our reference cohort, we used linear regression to account for age differences. We modeled PSDs for all participants in the reference cohort using log_2_(age), where the log transform accounts for larger developmental gains at younger ages. We then reconstructed PSDs for each reference cohort participant using the log_2_(age) of the paternal Dup15q syndrome participant and adding back model residuals for each participant.

### Peak frequency extraction

**Fig. 1:**
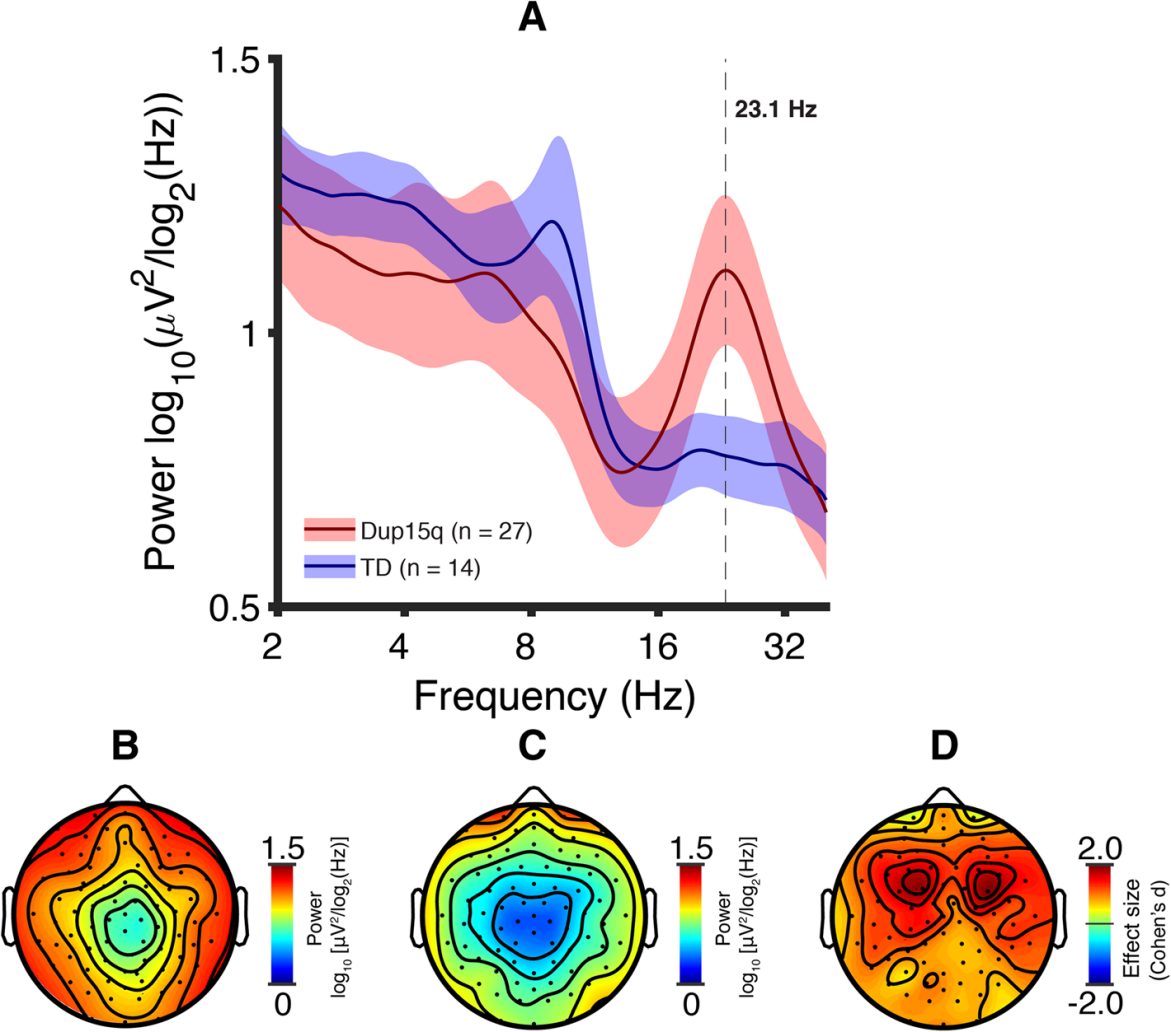
Dup15q syndrome versus TD. **a** Spectral profiles of children with Dup15q syndrome (red) and TD children (blue). PSDs are averaged across channels and participants; colored highlights represent 95% confidence intervals. Power is significantly higher in Dup15q syndrome at 20.2–28.5 Hz (*p* < 0.05 corrected). **b** Dup15q syndrome topographic scalp power (mean across participants at *f* = 23.1 Hz). **c** TD topographic scalp power (mean across participants at *f* = 23.1 Hz). **d** Dup15q syndrome versus TD power difference effect sizes (Cohen’s *d*) at *f* = 23.1 Hz. Mean effect size across channels, *d* = 1.06 (min, *d* = 0.339; max, *d* = 1.98)

**Fig. 2:**
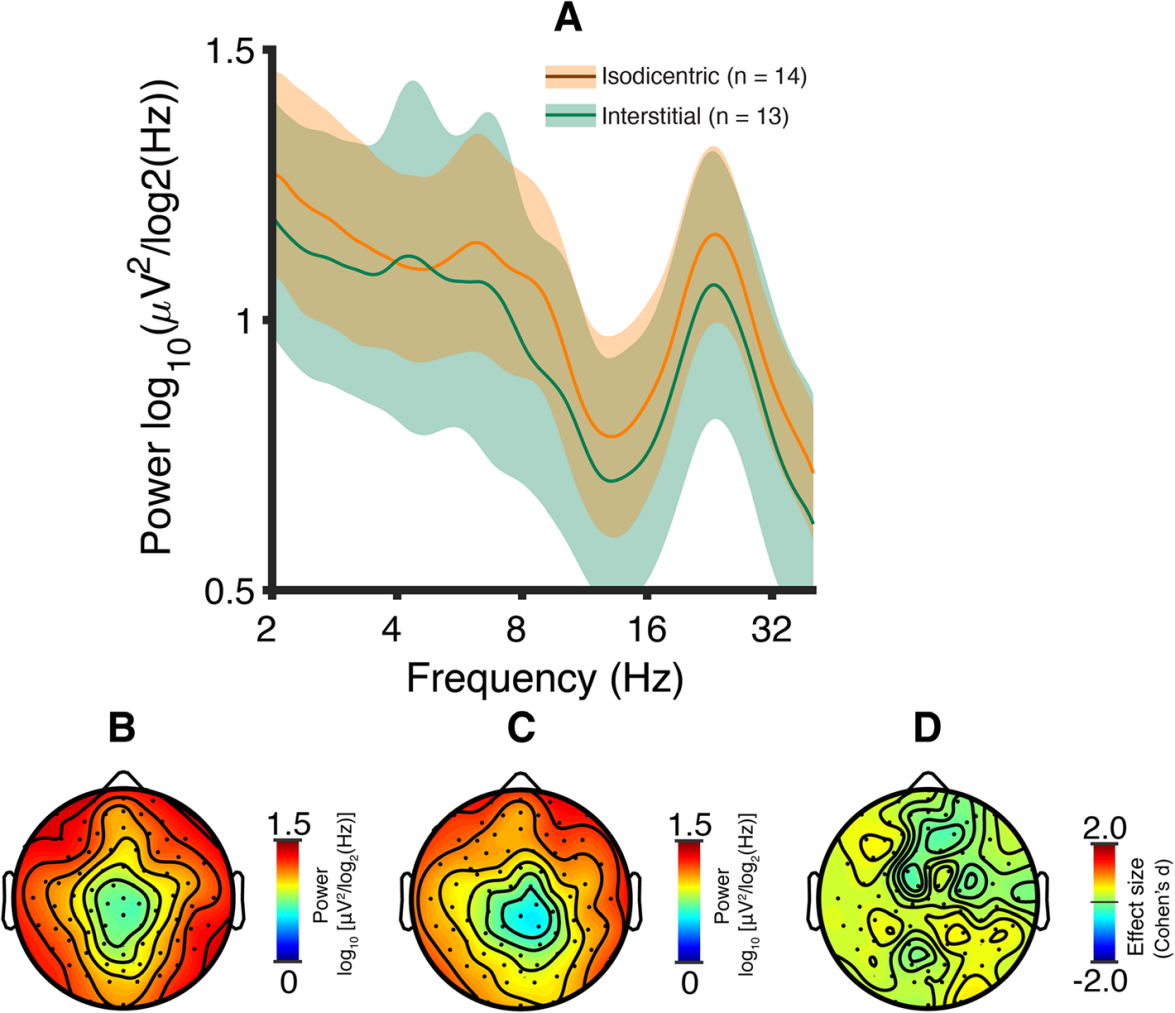
Dup15q syndrome by duplication type. **a** Spectral profiles of isodicentric (orange) and interstitial (green) duplications. PSDs are averaged across channels and participants; colored highlights represent 95% confidence intervals. Both duplication types show prominent spectral peaks in the beta band (group-level averages: isodicentric, *f* = 23.2 Hz; interstitial, *f* = 23.1 Hz). Power does not differ between duplication types (*p* > 0.05, all frequencies). **b** Mean topographic scalp power of participants with isodicentric duplications at *f* = 23.1 Hz (i.e., the Dup15q syndrome spline-interpolated peak frequency). **c** Mean topographic scalp power of all participants with interstitial duplications at *f* = 23.1 Hz. **d** Isodicentric versus interstitial power difference effect sizes (Cohen’s *d*) at *f* = 23.1 Hz. Mean effect size across channel, *d* = 0.21 (min, *d* = -0.19; max, *d* = 0.50)

To further investigate beta oscillations, we examined beta peak frequency in Dup15q syndrome (reference cohort and participants with paternal duplications). Identifying beta band peaks depends crucially on the presence of local maxima that are not smeared out by averaging across scalp regions with different peak frequencies. For this reason, our identification of beta peak frequency was done on power averaged only across frontal channels, as this scalp area featured the highest beta power in Dup15q syndrome (see Fig. 1 in the “Results” section, cf. Fig. 2 in Frohlich and colleagues 2016) [19]. HD EEGs were spatially interpolated to 19 channels corresponding to the international 10-20 montage and power was averaged across channels Fp1, Fp2, F3, F4, Fz, F7, and F8. Frontal beta peak frequency (FBPF) was automatically identified in each participant by extracting the beta band peak with the highest power.

## Results

### Dup15q syndrome reference cohort

We first compared PSDs, averaged across channels and participants, from the TD cohort and Dup15q syndrome reference cohort. The Dup15q syndrome reference cohort displayed a prominent group-level peak in the beta band (peak frequency: *f* = 23.1 ± 0.406 Hz, mean ± SEM), matching the peak frequency reported in a previous investigation [19]. Peak beta power did not differ between data collection sites for reference cohort participants (one-way ANOVA, *F*(2,24) = 0.40, *p* = 0.68, spline-interpolated peak frequency). We then compared EEG power at all frequencies between the Dup15q syndrome cohort and the TD cohort (two-tailed *t*-tests across 54 bins). We found elevated EEG power in Dup15q syndrome relative to TD children at 20.2–28.5 Hz (7 bins, *p* < 0.05 corrected using false discovery rate with the Benjamini-Hochberg procedure [45]). This finding confirms the presence of elevated beta power in Dup15q syndrome reported in previous work [19] using a larger cohort that includes some participants from the earlier study. Beta oscillations were observed globally across the scalp in Dup15q syndrome (Fig. 1b) compared to the TD cohort (Fig. 1c) at all channels (effect size: *d*′ = 1.06 ± 0.325, mean ± SD across channels). The largest effect sizes (*d* > 1) were located in frontocentral scalp regions (Fig. 1d).

To test for a gene dosage effect within Dup15q syndrome, we next evaluated channel-averaged PSDs separately for participants with interstitial and isodicentric duplications (Fig. 2a). We found no significant difference in beta power between duplication types at the Dup15q syndrome reference cohort peak frequency (*f* = 23.1 Hz, *p* = 0.25, *t* = 0.69, one-tailed *t*-test). Expanding our test to all frequency bins, we still detected no significant differences in power (two-tailed *t*-tests across 54 bins), even before correcting for multiple comparisons across frequency bins. We did, however, observe considerably larger variance in interstitial Dup15q syndrome at most frequency bins. Both duplication types featured prominent group-level oscillatory peaks in the beta band (interstitial peak frequency *f* = 23.1 ± 0.464 Hz; isodicentric peak frequency *f* = 23.2 ± 0.567 Hz, mean ± SEM). We also observed similar patterns of scalp topography for both duplication types at 23.1 Hz (Fig. 2b, c). Effect sizes of isodicentric power versus interstitial power at *f* = 23.1 Hz were small (Fig. 2d).

### The Dup15q syndrome beta EEG phenotype resembles the effects of pharmacological GABA_A_ modulation

**Fig. 3:**
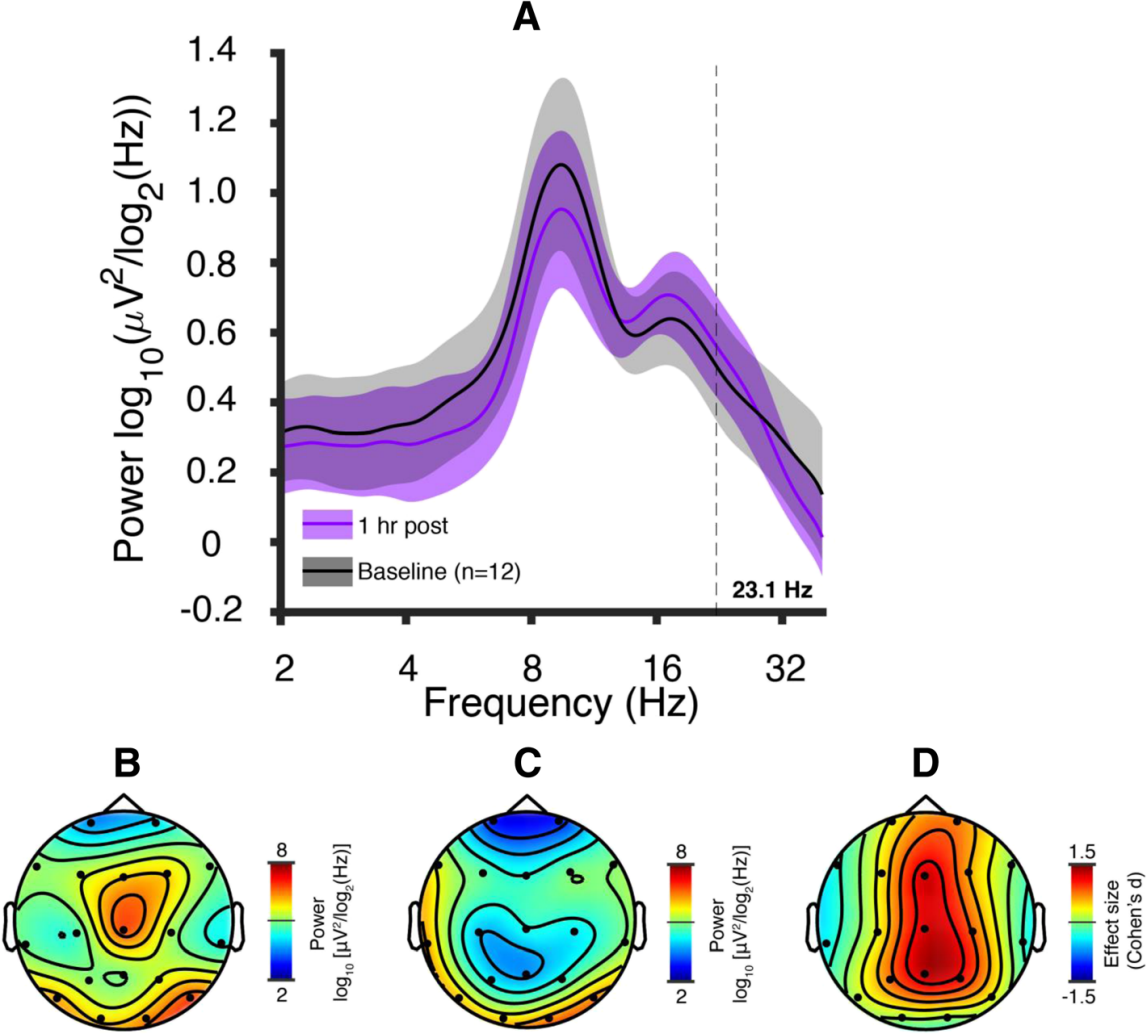
Midazolam pharmaco-EEG at baseline and 1 h post administration (5 mg oral). **a** PSDs averaged across participants for the baseline (black) and 1 h post administration (purple) conditions. Colored highlights represent 95% confidence intervals of the mean. Both conditions show spectral peaks in the alpha and beta bands; the alpha peak appears diminished and the beta peak appears enhanced by midazolam challenge. **b** Topographic scalp power 1 h post administration at the Dup15q syndrome peak frequency (23.1 Hz). **c** Topographic scalp power from the baseline condition at the Dup15q syndrome peak frequency (23.1 Hz). **d** Effect sizes (Cohen’s *d*) of midazolam-induced power change at 23.1 Hz. The largest power changes occur at central channels Fz, Cz, and Pz (Cf. Fig. 3c)

**Fig 4:**
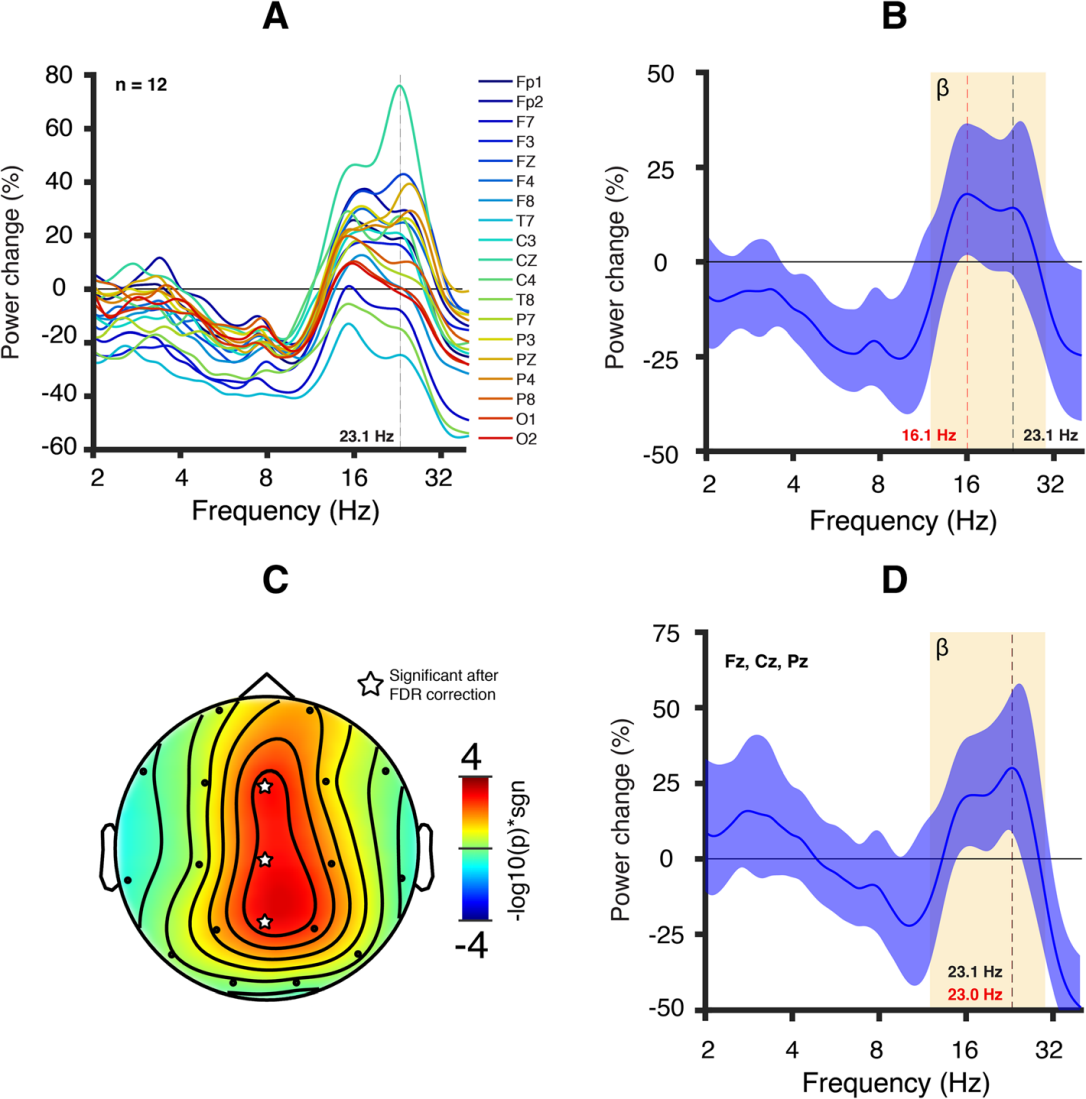
EEG signature of pharmacological GABA_A_ receptor modulation in healthy adult participants. Healthy adult participants (*n* = 12) were challenged orally with a GABA_A_ PAM (5 mg midazolam). **a** Average power change in all channels 1 h following drug administration referenced to baseline (absolute power averaged across participants). Most channels displayed an increase in power in the beta band. **b** Channel-averaged power change. The colored highlight represents the 95% confidence interval. The average power change appears to largely plateau between the peak power change (16.1 Hz, red vertical line) and the Dup15q syndrome peak frequency (23.1 Hz, black vertical line). **c** Scalp topography of the −log_10_(*p* value) multiplied by the sign of the *t*-statistic from a two-tailed *t* test at 23.1 Hz. Three central channels (Fz, Cz, and Pz, indicated with a star symbol) survive an FDR correction for multiple channels (*p* value threshold = 3 × 10^−3^). **d** Power change averaged across central channels. We visualized the average power change for those channels that survived the FDR correction at 23.1 Hz. The colored highlight represents the 95% confidence interval. The power change peaks at 23.0 Hz (red vertical line), very close to the Dup15q syndrome peak frequency (23.1 Hz, black vertical line; Cf. Fig. 1a).

Next, we compared the Dup15q EEG signature to the EEG signatures induced by a GABA_A_ PAM (midazolam, 5 mg oral administration) in healthy adult participants. The EEG showed spectral peaks in the alpha band and beta band both before and after midazolam administration (Fig. 3a). The midazolam condition showed the highest beta power in central scalp regions at the Dup15q syndrome peak frequency as compared with the baseline condition (*f* = 23.1 Hz, Fig. 3b–d). Most channels displayed positive changes in beta power, with the largest change occurring at channel Cz (76% increase, Fig. 4a, b). We then performed paired samples *t* tests across all channels at the Dup15q syndrome peak frequency. Three channels, Fz, Cz, and Pz, displayed a significant increase in power after correcting for multiple comparisons using the false discovery rate (FDR, Benjamini-Hochberg method, *p* < 0.05 corrected, Fig. 4c). The average power change for these channels yielded a peak frequency at 23.0 ± 1.61 Hz (mean ± SEM, 30% increase, Fig. 4d), very close to the Dup15q syndrome peak frequency (*f* = 23.1 ± 0.406).

### The beta EEG phenotype is observed in paternal Dup15q syndrome

Results from each participant with paternal Dup15q syndrome are described below separately. In both cases, we find that the quantitative beta EEG phenotype is observable in paternal Dup15q syndrome. This finding was observed qualitatively in an earlier publication [4].

### Paternal Dup15q participant 801-005

**Fig. 5:**
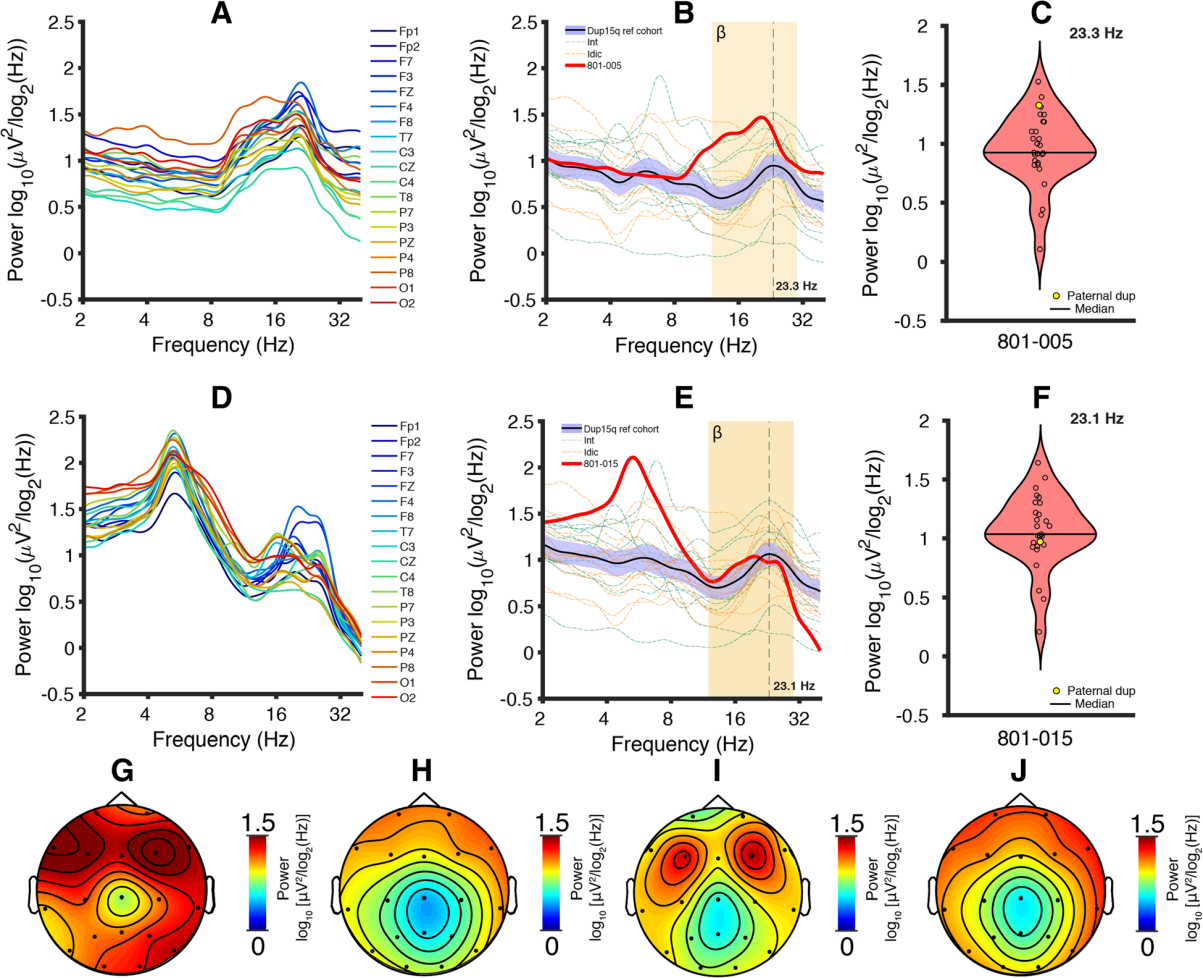
Paternal Dup15q syndrome PSDs and scalp topographies. **a** PSDs derived from all 19 channels (spatially interpolated from HD EEG) show prominent beta peaks in a 13-year-old boy with a paternal duplication (ID: 801-005). **b** Reference cohort PSDs were reconstructed using a simple linear regression model, plugging in the log age of 801-005 (161 months). Channel-averaged PSD derived from 801-005 shows higher beta power at all frequency bins than the Dup15q reference cohort 95% confidence interval of the mean. The channel-averaged peak beta frequency is lower in 801-005 (*f* = 20.4 Hz) as compared with the reference cohort (*f* = 23.3 Hz). **c** Violin plot of power at the reference cohort peak frequency (*f* = 23.3 Hz, reconstructed power), with 801-005 indicated in yellow near the top of the distribution. **d** PSDs derived from all 19 channels (clinical EEG) show prominent beta peaks in an 8-year-old girl with a paternal duplication (ID: 801-015). **e** Reference cohort PSDs were reconstructed using a simple linear regression model, plugging in the log age of 801-015 (96 months). Channel-averaged PSD derived from 801-015 shows beta power largely in the range of the Dup15q reference cohort 95% confidence interval. The channel-averaged peak beta frequency is lower in 801-015 (*f* = 19.7 Hz) as compared with the reference cohort (*f* = 23.1 Hz). **f** Violin plot of power at the reference cohort peak frequency (*f* = 23.1 Hz, reconstructed power), with 801-015 indicated in yellow near the mean of the distribution. **g** 801-005 beta power scalp topography measured at *f* = 23.3 Hz (reference cohort reconstructed power peak frequency). **h** Dup15q syndrome reference cohort beta power scalp topography measured at the group level peak frequency *f* = 23.3 Hz (reconstructed power from 801-005 regression model). **i** 801-015 beta power scalp topography measured at 23.1 Hz (reference cohort reconstructed power peak frequency). **j** Dup15q syndrome reference cohort beta power scalp topography measured at the group level peak frequency *f* = 23.1 Hz (reconstructed power from 801-015 regression model)

We observed highly prominent peaks in the beta band for PSDs derived from all examined channels from 801-005 (Fig. 5a, peak frequency: *f* = 19.8 ± 0.435 Hz, mean ± SEM). We then examined the channel averaged PSD in the context of the Dup15q reference cohort. Because of the broad age range of the reference cohort, we used a simple linear regression model to account for age differences (see the “Methods” section). Paternal duplication beta power was elevated above the Dup15q reference cohort 95% confidence interval of the mean for all beta frequencies (Fig. 5b). Similar results are yielded using only reference cohort participants with interstitial duplications (Additional file 1: Figure S2A). Beta power (reference cohort peak frequency) for 801-005 lies near the upper end of the reference cohort distribution (Fig. 5c, *f* = 23.3 Hz).

Next, we examined the topographic distribution of power distribution at the reference cohort peak frequency (reconstructed power). The scalp topography derived from 801-005 exhibited higher power at all channels (Fig. 5g) as compared with the reference cohort mean scalp topography (Fig. 5h). Scalp topography derived from only interstitial duplications in the reference cohort (Additional file 1: Figure S2C) appeared similar to that of the overall reference cohort. In all cases, we observed the highest power at frontal electrodes, also in line with previous findings by Frohlich and colleagues [19].

### Paternal Dup15q participant 801-015

We observed broadly elevated power across the beta band in PSDs derived from all examined channels in 801-015 (Fig. 5d, peak frequency: *f* = 19.3 ± 0.677 Hz, mean ± SEM) and peaks at multiple frequencies within the beta band (here we have reported the frequencies of the largest peaks). These factors cause smearing in the channel-averaged PSD, giving it a less prominent peak in the beta band than 801-005. Thus, the spectral profile of this paternal Dup15q syndrome case appears different than that of 801-015 while still exhibiting the beta EEG phenotype. We also observed highly prominent theta peaks for all channels in the 4–8 Hz frequency range (peak frequency: *f* = 5.29 ± 0.00275 Hz, mean ± SEM).

Beta power for 801-015 was higher than the Dup15q reference cohort 95% confidence interval from 13.8 to 19.5 Hz and within the confidence interval from 19.5 to 27.9 Hz, falling slightly below the mean at the reference cohort peak frequency (Fig. 5e, f). 801-015 showed a beta peak at *f* = 19.32 +/− 0.676 Hz (mean ± SEM), similar to the peak frequency observed for 801-005 (*f* = 19.82 +/− 0.435 Hz) and the reference cohort group-level peak frequency (*f* = 23.1 ± 0.406). Reference cohort participants with interstitial duplications also showed a group level peak frequency at 23.1 Hz (Additional file 1: Figure S2B).

The scalp topography (power at the Dup15 reference cohort peak frequency) derived from 801-015 exhibited power at 23.1 Hz comparable with the reference cohort mean scalp topography at the same frequency (Fig. 5i, j). Scalp topography was also similar between the overall Dup15q syndrome reference cohort and participants with interstitial duplications (Additional file 1: Figure S2D). 801-015 exhibited a bifrontal maximum in scalp power (channels at F3 and F4) at the reference cohort peak frequency; this is similar to the scalp topography seen in 801-005. Our findings indicate that beta power is elevated in individuals paternal Dup15q syndrome, strongly suggesting that overexpression of *UBE3A* is not necessary for the beta EEG phenotype

### Beta peaks in paternal Dup15q syndrome resemble those in Dup15q syndrome reference cohort

**Fig. 6:**
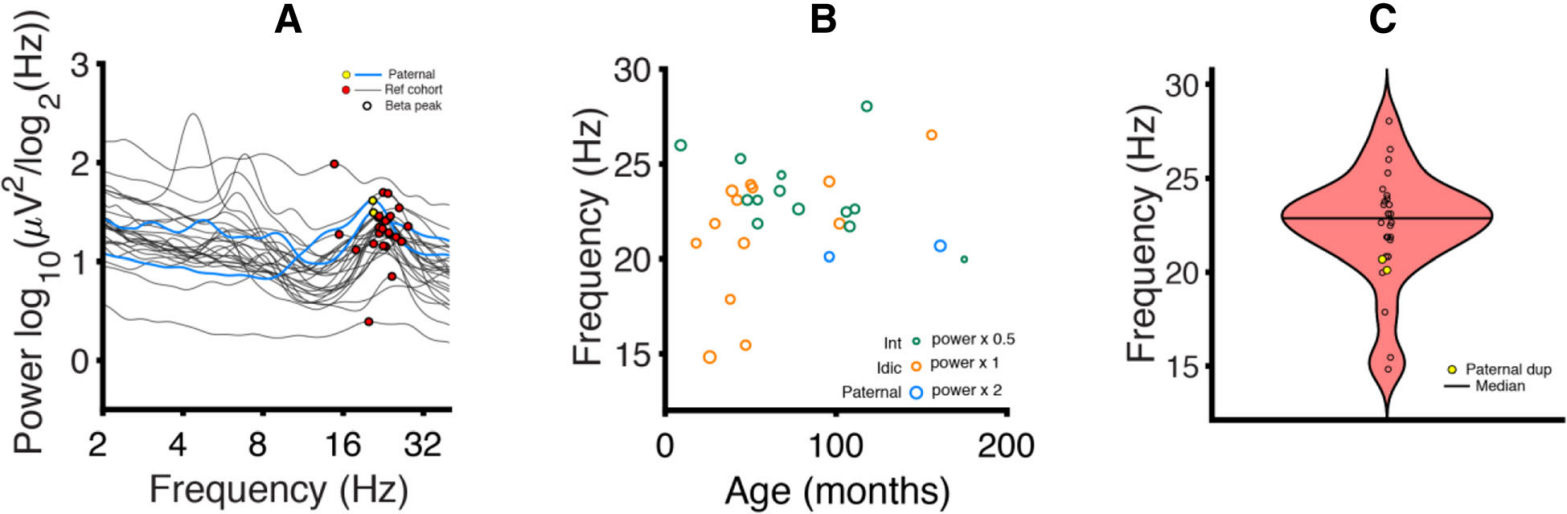
Peak frequency analysis. **a** PSDs derived from the Dup15q syndrome reference cohort (black) and paternal Dup15q syndrome (blue). Beta peaks are labeled in red (reference cohort) and yellow (paternal Dup15q). Both participants with paternal Dup15q syndrome appear to fall within the cluster of beta peaks found in the reference cohort. **b** Age versus FBPF. Points representing participants are sized proportionately to peak power at the FBPF (green = interstitial reference cohort, orange = isodicentric reference cohort, blue = interstitial paternal Dup15q syndrome). Age does not correlate with FBPF (*r* = 0.24, *p* = 0.25). **c** Violin plot of FBPF; yellow points represent paternal Dup15q syndrome. Both participants with paternal Dup15q syndrome fall within one standard deviation of the reference cohort

To further investigate the similarity of the maternal and paternal Dup15q syndrome beta-band oscillations, we investigated the FBPF for the Dup15q syndrome reference cohort (interstitial and isodicentric) and paternal Dup15q syndrome. Within the Dup15q syndrome reference cohort, we identified beta peaks in 26 out of 27 participants (96.3%, FBPF = 22.4 ± 2.99 Hz, mean ± SD, Fig 6a). We found that FBPF did not significantly relate to age in the reference cohort (*R*^2^ = 0.056, *p* = 0.25, Fig. 6b). For this reason, we did not implement regression modeling to age project the Dup15q syndrome reference cohort to the ages of paternal Dup15q syndrome participants. FBPF did not differ significantly between duplication types (interstitial vs isodicentric) within the reference cohort (*p* = 0.085, *t* = − 1.8). In paternal Dup15q syndrome, both participants had FBPF that fell within one standard deviation of the reference cohort mean (Fig. 6c; 801-005: FBPF = 20.7 Hz, *z* = − 0.59; 801-015: FBPF = 20.1 Hz, *z* = − 0.78 Hz; *z* scores are derived using the reference cohort mean and standard deviation). We also observed that participants with paternal Dup15q syndrome clustered well with the Dup15q syndrome reference cohort in frequency-power space (Fig. 6a).

## Discussion

There is a growing need in the field of neurodevelopmental disorders to identify scalable, mechanistic biomarkers that can directly improve clinical trials. With that goal, we have studied the properties of a robust EEG biomarker in Dup15q syndrome, one of the most common CNVs associated with ASD and ID. Here, we first replicated a prior study, with a larger cohort, and demonstrated that beta EEG power discriminates Dup15q syndrome from TD children. We then provided two lines of evidence that the Dup15q beta EEG phenotype likely reflects modulation of GABAergic neurotransmission. First, the Dup15q syndrome EEG signature resembles the EEG pattern found in pharmacological GABA_A_ receptor modulation of healthy adult participants. Second, the Dup15q syndrome EEG signature is present even in children who have presumably normal expression of *UBE3A* in cortical neurons (paternal duplications). By identifying a likely GABAergic mechanism underlying this phenotype, our work facilitates the application of this biomarker to clinical trials of drugs that target GABA for Dup15q syndrome, either as a pharmacodynamic biomarker or a response biomarker. Furthermore, our work informs future studies that may be used to rescue the beta EEG phenotype in animal models of Dup15q syndrome.

### GABA_A_ receptor modulation resembles the beta EEG phenotype in healthy adults

Our study is the first to compare the spectral EEG profile of healthy adults challenged with a benzodiazepine compound, midazolam, to the spectral EEG profile of Dup15q syndrome. Notably, several channels in the midazolam treatment group show nearly the same peak power change as the Dup15q syndrome peak frequency. This similarity of power spectral effects suggests that the Dup15q syndrome beta EEG phenotype might reflect GABA_A_ receptor dysfunction related to the dysregulation of the *GABRB3*/*GABRA5*/*GABRG3* gene cluster. However, so far, there is a lack of clear evidence for overexpression of these genes from Dup15q syndrome postmortem brain tissue studies [46–48]. Nonetheless, a vast body of prior literature demonstrates that GABA_A_ modulators such as benzodiazepines induce beta rhythms [33, 49–54] with similar spectral profiles to those observed in Dup15q syndrome [19], thus linking beta EEG activity to GABAergic activity. Interestingly, the topographic power distributions for Dup15q syndrome (Fig. 1b) and midazolam drug challenge (Fig. 3b) appear quite different; this may reflect the specific spatial distribution of affected β3/α5/γ3 subunits in Dup15q syndrome compared to the overall GABA_A_ receptor distribution that is modulated by the non-selective GABA_A_ PAM midazolam. In particular, the GABA_A_ receptor α5 subunit shows frontotemporal expression as revealed by PET studies with α5-subunit selective radioligands in humans [55–57] while GABA_A_ receptors targeted by non-selective benzodiazepines, such as midazolam, are widely and more homogenously distributed throughout the whole cortex. Finally, although a few channels (e.g., T7 and T8) showed decreased power in response to midazolam challenge in healthy adult participants, these channels also showed an overall frequency-nonspecific decrease in power (Fig. 4a), with local maxima in power change still occurring in the beta band.

### Elevated *UBE3A* is not necessary for the Dup15q syndrome EEG phenotype

Our data from two cases of paternal Dup15q syndrome, where *UBE3A* levels in most neurons are presumably normal, demonstrate that the beta EEG phenotype is not dependent on *UBE3A* alone. Instead, our data suggest that the beta EEG phenotype is dependent on one or more nonimprinted genes within the duplicated region. These findings are consistent with recent work in Angelman syndrome—a related 15q disorder—demonstrating reduced beta power in children with 15q11-q13 deletions compared to children with etiologies confined to *UBE3A* or other imprinted genes [27]. Although *UBE3A* is currently believed to be the only paternally imprinted gene in the 15q11.2-q13.1 region [58–60], our results strongly suggest that other genes on this locus that may also be paternally imprinted are unlikely to contribute to the Dup15q syndrome EEG phenotype.

Both paternal Dup15q syndrome cases were of different ages and studied with different EEG systems, thus suggesting that beta oscillations in paternal Dup15q syndrome are neither specific to one particular developmental age group nor an artifact of a particular EEG system. These results are consistent with a previous study qualitatively reporting that three out of four participants with paternal interstitial Dup15q syndrome exhibit the beta EEG phenotype [4]. EEG data from the remaining two participants in this earlier study were not available, and thus we were not able to quantitatively reexamine their EEG. Combining our findings from paternal Dup15q syndrome with those from our investigation of midazolam in healthy adults, we conjecture that GABAergic activity plays an important role in Dup15q syndrome cortical dynamics. Additional data from more patients with paternal Dup15q syndrome will be needed to support this conjecture.

### Towards quantitative biomarkers of neurodevelopmental disorders for drug development and clinical trials

Advances in genetic sequencing and testing have yielded an increasing proportion of ASD cases (3–20%) with a readily identifiable genetic cause [61, 62]. Duplications of 15q are the most common copy number variation identified in ASD, accounting for 1–3% of cases [9, 63]. Furthermore, polymorphisms [64–70] and abnormal expression levels [71–74] of several GABA_A_ receptor subunit genes have also been identified in individuals with ASD. In fact, single-nucleotide polymorphisms in all three of the GABA_A_ receptor genes that are duplicated in Dup15q syndrome were recently found to predict symptom-based and developmental deficits in a large (*n* = 99) cohort of children and adolescents with ASD [75]. At the same time, EEG beta band anomalies have been linked to ASD [76–79], though arguably less so than anomalies in other frequency bands [80]. It is possible beta activity in some individuals with nonsyndromic ASD reflects a genetic subtype with a GABAergic etiology, e.g., caused by point mutations in GABA_A_ receptor subunit genes.

The relationship between beta activity and ID is less clear, although much work has linked beta to attention and cognition [81–83]. However, the high-amplitude beta activity resulting from GABAergic dysfunction (e.g., in benzodiazepine drug challenge) may reflect different circuits and physiological processes than beta activity in the reports cited above, e.g., because benzodiazepines are associated with sedation rather than heightened attention [84]. In Angelman syndrome, a disorder highly penetrant for ID [85], beta power is reduced in cases caused by 15q11-q13 deletion relative to cases with etiologies that mainly impact UBE3A [27], suggesting a positive relationship between beta power and *GABRB3*/*GABRA5*/*GABRG3* copy number. This finding, combined with our findings herein (Fig. 4), suggest a GABAergic mechanism for the Dup15q syndrome beta EEG phenotype. Thus, beta activity in Dup15q syndrome may be functionally different than beta activity linked to attention and cognition in other populations.

Given that our study provides additional evidence that altered GABAergic signaling is a likely mechanism of the beta EEG phenotype in Dup15q syndrome, this phenotype may be used as a quantitative biomarker that reflects GABAergic dysfunction in Dup15q syndrome and other forms of ASD. Many clinical features of Dup15q syndrome are associated with altered excitatory/inhibitory balance, including ID [86, 87], ASD [88–90], and seizures [91, 92]. This motivates a clear readout of GABAergic tone in Dup15q syndrome. Moreover, it is known that children with Angelman syndrome have both a more severe clinical phenotype [34–37] and, as mentioned above, an altered beta EEG phenotype likely related to GABA_A_ receptor subunit genes [27]. Based on this observation, it is likely that beta power is directly related to GABAergic dysfunction and indirectly related to clinical phenotype in Dup15q syndrome and other neurodevelopmental disorders.

These putative GABAergic mechanisms underlying the Dup15q syndrome beta EEG phenotype open new doors to markers of pathophysiology and drug target engagement in Dup15q syndrome. Unlike the search for biomarkers in nonsyndromic ASD, here, we have identified an electrophysiological signature that has a plausible mechanism. Specifically, the molecular efficacy of drug treatments designed to correct excitatory/inhibitory balance in Dup15q syndrome by targeting GABA neurotransmission could be assessed using the beta biomarker, with changes in beta power or peak frequency serving as a robust marker of drug target engagement. Circuit changes that precede behavioral changes could also be measured using the beta biomarker, thus allowing investigators to evaluate the success of short trials that preclude observation of long-term behavioral changes. To this end, future work will explore the relationship between the beta EEG phenotype and clinical phenotypes in Dup15q syndrome using larger Dup15q syndrome cohorts including children with epilepsy who were excluded from this study.

### Limitations and future directions

We acknowledge several factors that often limit studies of rare conditions. (1) The healthy adult participants do not overlap in age with the Dup15q syndrome reference cohort, which is comprised entirely of children. (2) EEG data were acquired from healthy adults and children with Dup15q syndrome using different systems. These incongruencies between cohorts preclude a direct statistical comparison. (3) Furthermore, our conclusions are not formal inferences and do not prove that the mechanism underlying the Dup15q syndrome beta EEG phenotype is GABAergic. (4) Postmortem brain studies, limited by small samples, have yet to demonstrate a significant overexpression of the *GABRB3*/*GABRA5*/*GABRG3* gene cluster in Dup15q syndrome [46–48]. (5) Parent-of-origin data was not available for most reference cohort participants. However, because the *GABRB3*/*GABRA5*/*GABRG3* gene cluster is non-imprinted, the possible inclusion of paternal duplications in our reference cohort does not weaken our conclusions in any way. (6) Finally, although *UBE3A* is paternally silenced in most neurons, it is expressed biallelically in astrocytes [93]. This consideration may challenge the validity of paternal Dup15q syndrome as a *UBE3A*-normal control group. Nonetheless, the milder clinical phenotype of paternal Dup15q syndrome strongly suggests minimal *UBE3A*-related pathology in paternal duplications.

This work necessitates some future directions to confirm the promising conclusions drawn here. First, larger cohorts of children with paternal Dup15q syndrome should be examined with EEG, and this goal has prompted the development of a new pipeline, in partnership with the Dup15q Alliance, to upload and analyze data from clinically acquired EEGs in children with Dup15q syndrome. The role of *UBE3A* in EEG phenotype should also be examined in Prader-Willi syndrome, another 15q disorder caused by deletions/uniparental disomy of the paternal/maternal allele [94], the opposite of Angelman syndrome. In the future, we will examine Prader Willi syndrome to further disentangling the electrophysiological roles of *UBE3A* and *GABRB3*/*GABRA5*/*GABRG3*. Furthermore, future studies in patient-derived induced pluripotent stem cell cultures or Dup15q syndrome animal models should individually knockdown *GABRB3*, *GABRA5*, and *GABRG3*. Abolishing a Dup15q-like electrophysiological phenotype in these models through gene knockdown would demonstrate that one or more of these genes are necessary for the phenotype. Finally, we advocate for future studies exploring an expectedly milder beta EEG phenotype in cases of nonsyndromic ASD, in which GABAergic etiology is frequently implicated [9, 67, 95, 96].

## Conclusions

Quantitative biomarkers, rooted in mechanism and thus positioned to guide clinical trials, are greatly needed in neurodevelopmental disorders such as ASD. Here, we gained valuable insights into the mechanism of a robust EEG biomarker of Dup15q syndrome. This biomarker cannot be easily explained by elevated *UBE3A* levels per se but can be recapitulated by GABAergic modulation in healthy adults, suggesting that the phenotype might be a readout of increased GABA_A_ activity or sensitivity to GABA in Dup15q syndrome. Our work is an important step towards rooting the Dup15q syndrome biomarker in a molecular mechanism and facilitating its application in upcoming clinical trials.

## Additional file

Additional file 1: Supplementary material. (DOCX 944 kb)

## Data Availability

EEG data from Dup15q syndrome and TD control children are available from the corresponding author on reasonable request.

## Abbreviations

ADHD: Attention deficit hyperactivity disorder
ADOS: Autism diagnostic observation schedule
ASD: Autism spectrum disorder
Dup15q syndrome: Duplication 15q11.2-q13.1 syndrome
EGI: Electrical Geodesics, Inc
EEG: Electroencephalogram
FBPF: Frontal beta peak frequency
GABA_A_: Gamma-aminobutyric acid type-A
HD: High density
iPSC: Induced pluripotent stem cell
IRB: Institutional Review Board
PAM: Positive allosteric modulator
PSD: Power spectral density
TD: Typically developing
UCLA: University of California, Los Angeles
