# Correction to: Mechanisms underlying the EEG biomarker in Dup15q syndrome

**DOI:** 10.1186/s13229-019-0288-y

**Published:** 2019-11-06

**Authors:** Joel Frohlich, Lawrence T. Reiter, Vidya Saravanapandian, Charlotte DiStefano, Scott Huberty, Carly Hyde, Stormy Chamberlain, Carrie E. Bearden, Peyman Golshani, Andrei Irimia, Richard W. Olsen, Joerg F. Hipp, Shafali S. Jeste

**Affiliations:** 1 Roche Pharma Research and Early Development, Neuroscience, Ophthalmology and Rare Diseases, Roche Innovation Center Basel, Basel, Switzerland; 2 Center for Autism Research and Treatment, University of California Los Angeles, Semel Institute for Neuroscience, Los Angeles, CA 90024 USA; 3 Department of Psychology, University of California Los Angeles, 3423 Franz Hall, Los Angeles, CA 90095 USA; 4 Departments of Neurology, Pediatrics and Anatomy & Neurobiology, The University of Tennessee Health Science Center, 855 Monroe Ave., Link, Memphis, TN 415 USA; 5 McGill University, MUHC Research Institute, 5252, boul. de Maisonneuve Ouest, 3E.19, Montreal, QC H4A 3S5 Canada; 6 Genetics and Genome Sciences, UConn Health, 400 Farmington Avenue, Farmington, CT 06030-6403 USA; 7 Department of Psychiatry and Biobehavioral Sciences and Department of Psychology, University of California Los Angeles, Suite A7-460, 760 Westwood Plaza, Los Angeles, CA 90095 USA; 8 Department of Neurology and Psychiatry, David Geffen School of Medicine, 710 Westwood Plaza, Los Angeles, CA 90095 USA; 9 Leonard Davis School of Gerontology, University of Southern California, 3715 McClintock Ave., Suite 228C, Los Angeles, CA 90089 USA; 10 Department of Molecular and Medical Pharmacology, David Geffen School of Medicine at UCLA, Los Angeles, CA 90095 USA

**Correction to: Mol Autism (2019) 10:29**

**https://doi.org/10.1186/s13229-019-0280-6**

Following publication of the original article [1], we have been notified that the Ethics statement of the articles should be changed. The Ethics statement now reads:

All EEG studies and analyses were performed with institutional review board (IRB) and/or National Research Ethics Service approval.

The Ethics statement should read:

The EEG data collected for subject 801–015 was collected at University of Tennessee Health Science Centre (UTHSC) under an IRB approved protocol with informed consent, the transfer of this EEG data to University of California, Los Angeles (UCLA) for analysis was not approved by the UTHSC IRB. The data transfer was approved by the UCLA IRB with the understanding that the data file was de-identified, however, patient identifiers were included on the EEG disk in error. The EEG data for this subject are now stored on the UCLA server fully de-identified. All other subjects were collected at UCLA under an IRB approved protocol. The data and results presented in this article remain unaffected, and this correction has been published at the request of the Research Integrity Officer at UTHSC.

The authors gratefully acknowledge the support of the Economic & Social Research Council (award ES/P009255/1, Sustainable Care: connecting people and systems, 2017–21, Principal Investigator Sue Yeandle, University of Sheffield). Prof Mark Hawley is a Theme Lead for the National Institute for Health Research (NIHR) Devices for Dignity MedTech Co-operative (MIC). The work of the Devices for Dignity MIC is funded by the NIHR. The views expressed are those of the authors and not necessarily those of the NHS, the NIHR or the Department of Health and Social Care.
