# Supplementary material for: Mechanisms underlying the EEG biomarker in Dup15q syndrome
Source: Mol Autism. 2019 Jul 3;10:29. doi: 10.1186/s13229-019-0280-6 (PMC6609401; doi:10.1186/s13229-019-0280-6)
Supplement: Supplementary file 1 — Supplementary material. (DOCX 944 kb) [file 13229_2019_280_MOESM1_ESM.docx]

**Supplemental Information**

**Mechanisms underlying the EEG biomarker in Dup15q syndrome**

Joel Frohlich^1,2,3*^, Lawrence T. Reiter^4^, Vidya Saravanapandian^2^, Charlotte DiStefano^2^, Scott Huberty^2,5^, Carly Hyde^2^, Stormy Chamberlain^6^, Carrie E. Bearden^7^, Peyman Golshani^8^, Andrei Irimia^9^, Richard W. Olsen^10^, Joerg F. Hipp^1†^, Shafali S. Jeste^2†^

^1^Roche Pharma Research and Early Development, Neuroscience, Ophthalmology and Rare Diseases, Roche Innovation Center Basel, Switzerland

^2^Center for Autism Research and Treatment, University of California Los Angeles, Semel Institute for Neuroscience, Los Angeles, CA 90024, USA

^3^Department of Psychology, University of California Los Angeles, 3423 Franz Hall, Los Angeles, CA 90095, USA

^4^Deparetments of Neurology, Pediatrics and Anatomy & Neurobiology, The University of Tennessee Health Science Center 855 Monroe Ave., Link 415 Memphis, TN USA

^5^McGill university, MUHC Research Institute, 5252, boul. de Maisonneuve Ouest, 3E.19, Montreal, QC, H4A 3S5 Canada

^6^Genetics and Genome Sciences, UConn Health, 400 Farmington Avenue
Farmington, CT 06030-6403, USA

^7^Department of Psychiatry and Biobehavioral Sciences and Department of Psychology, University of California Los Angeles, Suite A7-460, 760 Westwood Plaza, Los Angeles , CA 90095 USA

^8^Department of Neurology and Psychiatry David Geffen School of Medicine 710 Westwood Plaza Los Angeles, CA 90095 USA

^9^Leonard Davis School of Gerontology, University of Southern California, 3715 McClintock Ave., Suite 228C, Los Angeles, California 90089, USA

^10^Department of Molecular and Medical Pharmacology, David Geffen School of Medicine at UCLA, Los Angeles, California 90095, USA

*Correspondence to: [joelfrohlich@gmail.com](mailto:joelfrohlich@gmail.com)

† Joint senior-authorship

**Supplemental Methods and Materials**

**Dup15q syndrome reference cohort**

Our reference cohort drew from a large sample of awake-state spontaneous EEG recordings collected from n = 70 participants with Dup15q syndrome as part of an ongoing study that included EEG and behavioral assessments. This sample encompassed children with both duplication types (interstitial and isodicentric) from three different sites: (1) UCLA, (2) the 2015 National Dup15q Alliance conference in Orlando, Florida, and (3) the 2017 National Dup15q Alliance conference in Redondo Beach, California.

For this study, we considered only children (age < 18 years) with full 15q11-q13 duplications (duplications of only BP1-BP2 were excluded). Participants with confirmed paternal duplications were excluded from the reference cohort, leaving only children with maternal or unknown parent-of-origin. Several participants gave data at multiple visits. In these instances, we processed EEG data from the earliest visit with usable data. To eliminate confounding factors such as epilepsy and antiepileptic medications that act on GABAergic transmission, we carefully selected a subsample of children with no history of seizures and no antiepileptic medications of any type. After eliminating n = 40 participants due to medication, seizures, or data quality, n = 2 further participants were excluded for the following reasons: 1) sleep during EEG recording, and 2) fewer than 20 valid time windows for the lowest frequency analyzed. An additional (n = 1) participant with paternal Dup15q syndrome was removed from the reference cohort and examined separately. Our final reference cohort retained n = 27 participants (UCLA, n = 8; Orlando, n = 7; Redondo, n = 12). Of these participants, n = 13 were children that also gave EEG data included in a within group analysis of Dup15q syndrome beta power from a prior study [1]. A smaller subset (n = 5) of these children were also included in a between groups analysis of Dup15q syndrome beta power with TD and non-syndromic ASD controls.

**Paternal Dup15q syndrome case studies**

We obtained HD EEG from a 13-year-old boy with paternal Dup15q syndrome (801-005) through UCLA according to the same protocol as the reference cohort. This EEG was also included in the within group analysis of Dup15q syndrome beta power by Frohlich and colleagues [1]. We also obtained clinical EEG from an 8-year-old girl with paternal Dup15q syndrome (801-015) seen at LeBonheur Children’s Hospital (LCH) in Memphis, Tennessee and recruited through the University of Tennessee Health Science Center (UTHSC). Neither paternal Dup15q syndrome participant had a history of seizures, though both participants had a diagnosis of attention deficit hyperactivity disorder (ADHD). See Table 2 for clinical phenotype and duplication size of both participants.

EEG data were collected from 801-015 at LCH in a multi-hour, nonconsecutive recording (sampling rate f = 512 Hz) using a 21-channel clinical system. We extracted 10 minutes of data from the longest segment of consecutive data in the recording and excluded ear channels (A1 and A2), retaining 19 channels corresponding to the 10-20 montage. Data were referenced to average prior to importing. Clinical EEGs were performed using a Nicolet EEG machine with Pz reference. Because the number of EEG channels differed between recording systems used for each participant, EEG data from the participant with the greater number of EEG channels (801-005) were spatially interpolated to a 19-channel montage congruent with the other participant’s EEG.

**Midazolam pharmaco-EEG**

We examined the EEG from n = 12 healthy adult controls challenged with the benzodiazepine compound midazolam. The study protocol was approved by the National Research Ethics Service (NRES) committee. A baseline EEG was recorded at the start of the study at the same time of day that the drug would be administered at a later date. A sub-sedative dose of midazolam was administered orally (5 mg) on day 2 of the study, and pharmaco-EEG was recorded 1 hour following drug challenge (4 minutes eye closed resting, 4 minutes eyes open resting). On both days, EEG data were acquired at sampling rate of 256 Hz (alpha trace digital EEG EPV-32, Neuro Medical, Arnheim, Netherlands; recording reference POz). We averaged spectral power across eyes open and eyes closed data within each condition (baseline and post drug) following a wavelet frequency transform.

**Figures and analysis**

For generating figures and to precisely report peak frequencies, we used a spline-interpolation to derive a higher frequency resolution (100 frequency bins per octave). Peak frequencies are reported as the local maximum in the beta band (14 – 30 Hz, lower frequencies are excluded to avoid spectral leakage from the alpha band). Standard error of the mean (SEM) was computed for peak frequencies across participants using a jackkniefing approach. In instances of multiple beta peaks, only the largest peak is captured by the local maximum. Topographic scalp plots were generated using EEGLAB for HD EEG [2] and Fieldtrip [3] for 19-channel EEG. Violin plots were generated using code by Hoffmann [4]. Power differences were tested with *t*-tests using the Satterthwaite approximation to derive corrected degrees of freedom in cases of unequal variances.

**Peak frequency extraction**

To further analyze beta oscillations, we examined beta peak frequency in Dup15q syndrome (reference cohort and participants with paternal duplications). Identifying beta band peaks depends crucially on the presence of local maxima that are not smeared by averaging across scalp regions with different peak frequencies. For this reason, our identification of beta peak frequency was done on power averaged only across frontal channels, as this scalp area featured the highest beta power in Dup15q syndrome (Fig. 1, cf. Fig.2 in Frohlich and colleagues 2016) [1]. HD EEGs were spatially interpolated to 19 channels corresponding to the international 10-20 montage and power was averaged across channels Fp1, Fp2, F3, F4, Fz, F7, and F8. Frontal beta peak frequency (FBPF) was automatically identified in each participant after increasing frequency resolution to 100 frequency bins per octave (resolution increased by a factor of 735%) using a spline interpolation to derive accurate measurements. We then used the findpeaks() function in MATLAB to extract the beta band (12 -30 Hz) peak with the highest power. Specifically, peaks were defined as having 1) a minimum width of 10 frequency binds (1/10 octave) referenced to a vertical position a 1/2 of the peak prominence and 2) a minimum prominence (vertical drop) of 0.025 log_10_(μV^2^/octave). We extracted at most one peak per participant.

**Supplemental Tables**

|  | Clean data | Bad channels | Total channels | Artifact ICs | Total ICs |
| --- | --- | --- | --- | --- | --- |
| Reference cohort | 217 ± 121 s | 1.56 ± 2.08 | 83 | 33.1 ± 12.5 | 82 |
| TD controls | 128 ± 31.9 s | 0.86 ± 1.17 | 83 | 22.6 ± 8.75 | 82 |
| Paternal dup 801-005 | 483 s | 0 | 19 | 34 | 82 |
| Paternal dup 801-015 | 581 s | 0 | 19 | 2 | 18 |
| Healthy adult cohort | 221 ± 26.7 s | 0 ± 0 | 19 | 4.9 ± 1.94 | 18 |

***Table S1 Details of data processing.*** *Second of clean data, number of bad channels, and number of artifact components are given as mean ± SD for datasets analyzed from the reference cohort, TD controls, and healthy adult cohort; the total number of each category is also reported for both paternal duplication cases. Each dataset analyzed had a minimum of 60 s of clean data. Datasets for which the number of bad channels exceeded the square root of the total number of channels were discarded and not analyzed. Numbers for the healthy adult cohort are reported as mean and standard deviation per participant, and drug condition.*

**Supplemental Figures**


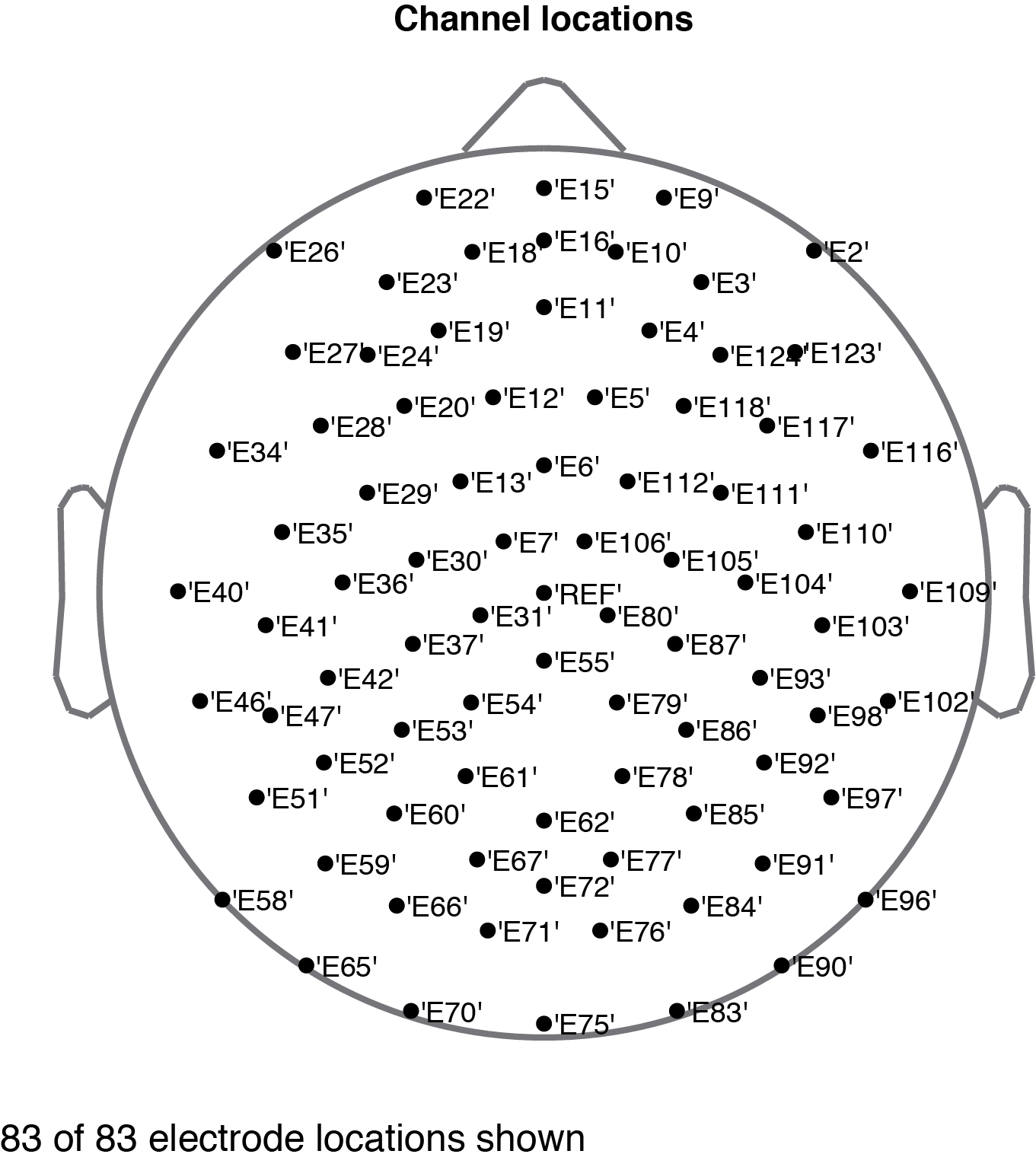


***Figure S1 HD EEG channels used in analysis.*** *For all EEG collected through UCLA using 129 channel EGI geodesic nets (“HD EEG”), we excluded 46 “skirt channels” (defined as channels with EGI polar coordinate r > 0.5) near the periphery of the EEG net that are particularly sensitive to noise and muscle artifact. The remaining 83 channels retained for analysis are shown above and labeled with their EGI channel number.*


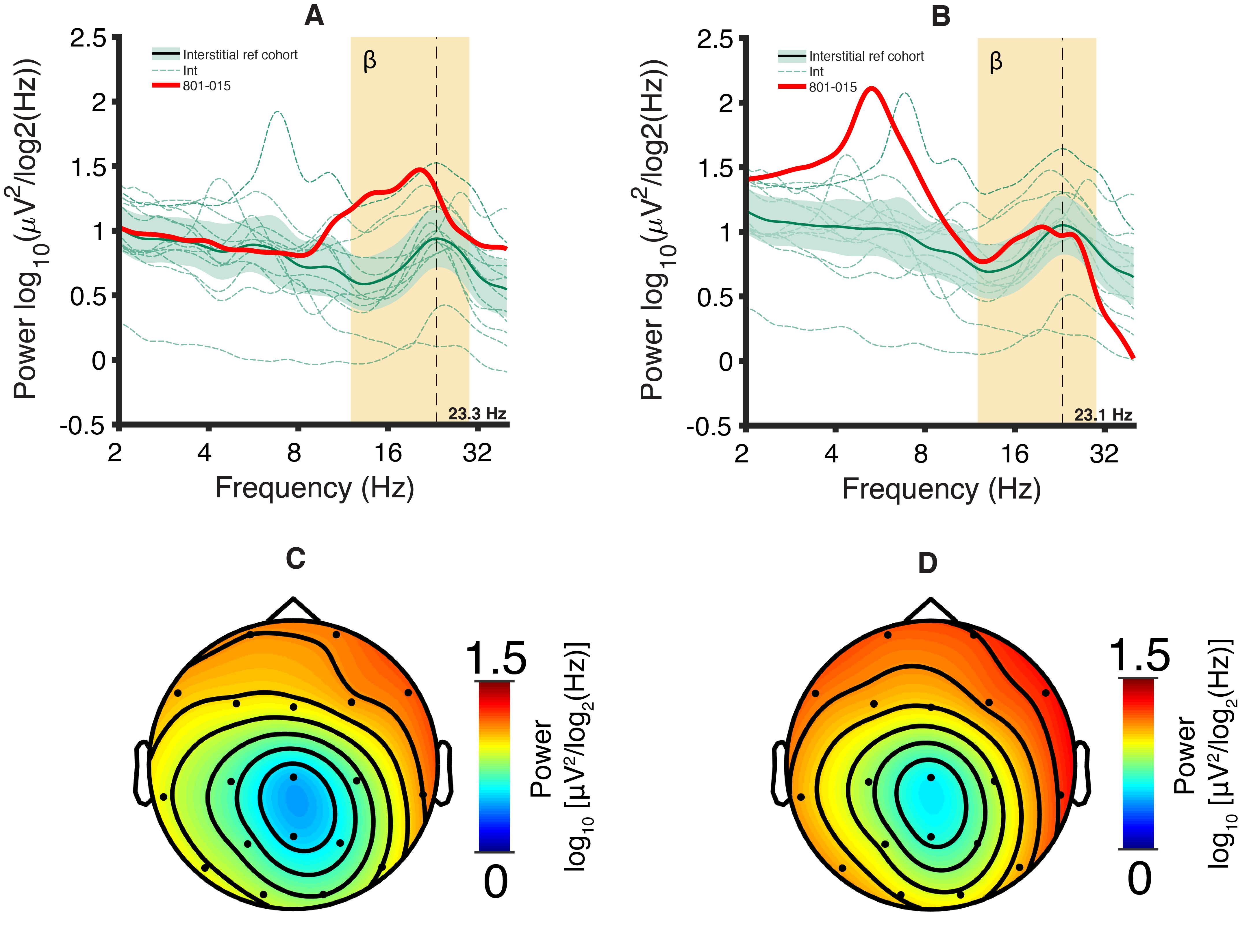


***Figure S2 Paternal Dup15q syndrome PSDs compared with the interstitial Dup15q syndrome reference cohort.*** *(A) Channel-averaged PSD derived from 801-005 compared with the interstitial reference cohort. 801-005 shows higher power at most beta frequencies than the interstitial reference cohort 95% confidence interval. (B) Channel-averaged PSD derived from 801-015 compared with the interstitial reference cohort. 801-015 shows beta power almost entirely in the range of the interstitial reference cohort 95% confidence interval. (C) Interstitial reference cohort beta power scalp topography measured at the group level peak frequency f = 23.3 Hz (reconstructed power from 801-005 regression model). (D) Interstitial reference cohort beta power scalp topography measured at the group level peak frequency f= 23.1 Hz (reconstructed power from 801-015 regression model).*

**Supplemental References**

1. Frohlich J, Senturk D, Saravanapandian V, Golshani P, Reiter LT, Sankar R, et al. A quantitative electrophysiological biomarker of duplication 15q11. 2-q13. 1 syndrome. PloS One. 2016;11:e0167179.

2. Delorme A, Makeig S. EEGLAB: an open source toolbox for analysis of single-trial EEG dynamics including independent component analysis. J Neurosci Methods. 2004;134:9–21.

3. Oostenveld R, Fries P, Maris E, Schoffelen J-M. FieldTrip: open source software for advanced analysis of MEG, EEG, and invasive electrophysiological data. Comput Intell Neurosci. 2011;2011:1.

4. Hoffmann H. Simple violin plot using matlab default kernel density estimation. [Internet]. Katzenburgweg 5, 53115 Germany: INRES (University of Bonn); 2015. Available from: https://ch.mathworks.com/matlabcentral/fileexchange/45134-violin-plot
